# IGF2BP2 Induces U251 Glioblastoma Cell Chemoresistance by Inhibiting FOXO1-Mediated PID1 Expression Through Stabilizing lncRNA DANCR

**DOI:** 10.3389/fcell.2021.659228

**Published:** 2022-01-24

**Authors:** Junfei Han, Xiaojun Yu, Shanxi Wang, Yingguang Wang, Qikun Liu, Haoran Xu, Xiaosong Wang

**Affiliations:** ^1^ Department of Neurosurgery, Huizhou Third People’s Hospital, Huizhou Hospital Affiliated to Guangzhou Medical University, Huizhou, China; ^2^ Department of Orthopedics, Tongji Hospital, Tongji Medical College, Huazhong University of Science and Technology, Wuhan, China; ^3^ Department of Neurosurgery, The First Hospital of Qiqihar, Qiqihar, China; ^4^ Department of Neurosurgery, Affiliated Qiqihar Hospital, Southern Medical University, Qiqihar, China

**Keywords:** glioma, drug resistance, glioblastoma, IGF2BP2, DANCR, FoxO1, etoposide

## Abstract

Glioma is the most common type of malignant tumor of the nervous system and is characterized by high mortality and poor outcome. This study aims to investigate the mechanism underlying IGF2 mRNA-binding protein 2 (IGF2BP2) and long noncoding RNA DANCR in etoposide resistance of glioblastoma (GBM) cells. Bioinformatics analysis identified the IGF2BP2-related regulators and DANCR target genes, which were subsequently evaluated by RNA pull-down and RIP assays. We exposed GBM cells to etoposide and thus established etoposide-resistant cells. Through functional experiments, we evaluated the interrelationship among IGF2BP2, DANCR, phosphotyrosine interaction domain containing 1 (PID1), and forkhead box protein O1 (FOXO1) and further assessed their impact on the sensitivity of GBM cells to etoposide. IGF2BP2 and DANCR were highly expressed in glioma cells and tissues, whereas PID1 and FOXO1 were poorly expressed. Mechanistically, overexpression of IGF2BP2 promoted DANCR stability and reduced DANCR methylation, whereas silencing of IGF2BP2 reduced survival of GBM cells and etoposide-resistant cells. Besides, DANCR interacted with FOXO1 to promote the ubiquitination of FOXO1. FOXO1 promoted the transcriptional expression of PID1, enhancing the chemotherapy sensitivity of GBM cells, but overexpression of PID1 reversed the impact of IGF2BP2. Collectively, IGF2BP2 inhibits PID1 expression through the DANCR/FOXO1 axis, inducing drug resistance in GBM cells, and promoting glioma progression.

## Introduction

Brain tumors are commonly occurring solid tumors that cause significant cancer-related mortality (McNeill, 2016). Glioma originates from the glial cells in the brain and spinal cord and can be graded on a I–IV scale based on morphology and malignant behavior specified in the World Health Organization (WHO) classification, (Rasmussen et al., 2017; Cai et al., 2020). Glioblastoma (GBM), at WHO grade IV, is the most malignant and aggressive version of glioma in adults (Gusyatiner and Hegi, 2018; Lee et al., 2018). With the exception of temozolomide, GBM is relatively unresponsive to most chemotherapy agents (Tan et al., 2020). Therefore, it is imperative to clarify the mechanisms of resistance to etoposide, aiming to develop an improved chemotherapy for glioma.

In the presence of a given drug, epigenomes acting through aberrant transcription of drug transporters, DNA-repair enzymes, and proapoptotic factors can progressively render the cytotoxic and targeted drugs ineffective and allow the selection of rare drug-resistant tumor cell lines (Wilting and Dannenberg, 2012). Previous studies have revealed the role of specific factors in drug resistance to etoposide in glioma, such as X-linked inhibitor of apoptosis (XIAP) (Lopez et al., 2012), integrin β3 (Kim et al., 2011), and others. Apart from drug resistance, the blood–tumor barrier (BTB) is characterized by numerous distinct features, which can significantly attenuate the efficacy of chemotherapy for glioma (Arvanitis et al., 2020). Knockdown of IGF2 mRNA-binding protein 2 (IGF2BP2) is noted to decrease the expression of long noncoding RNAs (lncRNAs) and tight junction–related proteins, thereby promoting BTB permeability in glioma and enhancing antitumor efficacy of certain drugs (Liu et al., 2020). IGF2BP2 is known to participate in various biological processes, including development, tumorigenesis, and stemness, while also mediating posttranscriptional regulation of RNAs (Dai, 2020). IGF2BP2 (also known as Imp2) activates the PI3K/Akt signaling pathway and thereby promotes glioma progression, whereas its inhibition sensitizes glioma cells to temozolomide treatment (Mu et al., 2015). However, the mechanism underlying the role of IGF2BP2 in glioma resistance remains elusive, and its association with lncRNAs thus merits further attention.

LncRNAs have been highlighted to induce chemoresistance of cancer cells through a variety of factors including regulation of drug efflux, DNA damage repair, cell cycle, apoptosis, epithelial–mesenchymal transition, induction of signaling pathways, and angiogenesis (Majidinia and Yousefi, 2016; Qu et al., 2020). In glioma, some lncRNAs are highlighted as a promising therapeutic strategy to reduce drug resistance. For example, the knockdown of lncRNA MIR155HG increases glioma sensitivity to temozolomide through inactivation of Wnt/β-catenin pathway (He et al., 2020). Conversely, the lncRNA DANCR promotes cisplatin resistance by activating the AXL/PI3K/Akt/nuclear factor κB signaling pathway in glioma (Ma et al., 2018). Notably, DANCR may be a good target for the treatment of glioma patients, as its normal activity can contribute to malignancy and poor prognosis of glioma (Xu et al., 2018; Yang et al., 2018). Interestingly, DANCR is modified at N6-methyladenosine (m6A), whereas IGF2BP2 serves a reader for m6A-modified DANCR and stabilizes DANCR RNA in pancreatic cancer (Hu et al., 2020). Based on the documented roles of DANCR and IGF2BP2 in glioma and their correlation in certain cancers, we aimed in this study to explore the interaction between DANCR and IGF2BP2 in drug resistance in GBM cells. Transcription factor forkhead box protein O1 (FOXO1) is located at a convergence point of receptor tyrosine kinase signaling and is also highlighted to be associated with sensitivity to chemotherapy drugs against glioma (Tang et al., 2016; Chen et al., 2019). FOXO1 upregulates the expression of the protein phosphotyrosine interaction domain containing 1 (PID1), thereby inhibiting the tumorigenicity and growth of glioma stem cells (Zhao et al., 2017). In the current experiments, we set about to examine the regulatory effects that IGF2BP2 on GBM cell chemoresistance and analyze the potential regulatory network of the IGF2BP2/DANCR/FOXO1/PID1, aiming to provide a novel antitumor target for tumor treatment.

## Methods

### Ethics Statement

The current study was approved by the ethics committee of the First Hospital of Qiqihar and Affiliated Qiqihar Hospital, Southern Medical University, and performed in strict accordance with the Declaration of Helsinki. All participants or caregivers signed informed consent documentation before sample collection. Animal experiments were approved by the Animal Ethics Committee of the First Hospital of Qiqihar and Affiliated Qiqihar Hospital, Southern Medical University, and strictly performed according to the Guide for the Care and Use of Laboratory Animals published by the US National Institutes of Health. Extensive efforts were made to ensure minimal suffering of the included animals.

### Clinical Sample Collection

Glioma tissue samples were surgically obtained from 40 patients with glioma at the First Hospital of Qiqihar and Affiliated Qiqihar Hospital, Southern Medical University, from June 2017 to June 2019. Normal brain tissues were collected as control material from 40 patients with traumatic brain injury. None of the glioma patients had received antitumor treatment before surgery. One portion of the harvested tissues was immersed in formalin for subsequent immunohistochemical analysis, and the other part was directly transferred to cryotubes (−80°C) for storage.

### Cell and Reagents

GBM cell lines U251MG (namely, U251), LN229, LN18, and T98G were purchased from EK-Bioscience Co., Ltd. (Shanghai, China). The primary normal human astrocytes NHA and human embryonic kidney 293 (HEK293T) cells were purchased from BLUEFBIO Co., Ltd. (Shanghai, China). GBM cells and NHA cells were cultured in Dulbecco modified eagle medium (DMEM; Gibco, Grand Island, NY, United States). HEK293T cells were cultured in minimum essential medium (Gibco) containing 10% fetal bovine serum (Gibco), 2 mM l-glutamine (Sigma–Aldrich, Chemical Company, St. Louis, MO, United States), 100 U/mL penicillin, and 100 μg/mL streptomycin (Gibco) at 37°C, and 5% CO_2_. Actinomycin D and cycloheximide (CHX) were purchased from Sigma–Aldrich, St. Louis, MO, United States; MG132 was purchased from Yeasen Biotechnology (Shanghai, China), and etoposide was purchased from Selleck (Houston, TX, United States).

### Cell Treatment

The firefly luciferase Luc2P was cloned into the polyclonal sites of pHAGE-CMV-MCS-IRES-ZsGreen plasmid and pSUPER-retro-puro-Luc vector to construct pHAGE-puro-Luc and pSUPER-retro-puro-Luc vectors. pHAGE-puro-Luc vector was used to establish overexpression plasmids: pHAGE-IGF2BP2 (IGF2BP2 overexpression), pHAGE-DANCR (DANCR overexpression), pHAGE-S1m-DANCR (S1m-modified DANCR overexpression), pHAGE-FOXO1 (FOXO1 overexpression), and pHAGE-PID1 (PID1 overexpression); pSUPER-retro-puro-Luc vector established interference plasmids with short hairpin RNA (shRNA) oligonucleotide sequence: pSUPER-sh-IGF2BP2 (IGF2BP2 shRNA), pSUPER-sh-DANCR (DANCR shRNA), pSUPER-sh-FOXO1 (FOXO1 shRNA), and pSUPER-sh-PID1 (PID1 shRNA).

shRNA oligonucleotide sequence was cloned into the pSUPER-retro-puro-Luc vector (Invitrogen Biotechnology, Shanghai, China) for RNA knockdown experiments and the shRNA sequences targeting each gene (as well as NC for shRNA sequences) were synthesized by Invitrogen. The specific sequences are shown in Supplementary Table S1.

RNA knockdown experiments: U251 cells were classified into groups and transduced with lentivirus carrying shRNA-negative control (sh-NC) + vector, sh-IGF2BP2 + vector, sh-NC + DANCR, sh-IGF2BP2 + DANCR, vector, DANCR, FOXO1, DANCR + FOXO1, PID1, FOXO1 + sh-NC, vector + sh-PID1, FOXO1 + sh-PID1, IGF2BP2, and IGF2BP2 + PID1, respectively, using Lipofectamine 2000 reagent (Invitrogen, Carlsbad, CA, United States).

Using Lipofectamine 2000 reagent (Invitrogen), the lentiviral vector pHAGE series plasmids and adjuvant plasmids pSPAX2 and pMD2.G were transduced into HEK293T cells, and 72 h later, the supernatant was harvested to obtain the overexpression plasmid series lentiviral solution. The retroviral vector pSUPER series plasmids and adjuvant plasmids gag/pol and VSV.G were transfected into HEK293T cells, and 72 h later, the supernatant was collected to obtain the shRNA plasmid series retroviral solution. U251 cells were transduced with the lentivirus-carried overexpression plasmids. According to the fluorescence, flow cytometry was used to sort the stably transduced overexpression cell line. Thereafter, the stably overexpressed cell lines were transduced with retrovirus-carried shRNA plasmids or overexpression plasmids. After 72 h, 4 μg/mL puromycin was added for selecting the stably transduced cell lines.

### Construction of Etoposide-Resistant Cell Lines

U251 cells at logarithmic growth phase were digested, seeded on plates, and cultured for 24 h. Then the cells were transferred to a medium containing 2.5 μg/mL of etoposide (initially 1/50 of the parent cell line IC_50_). Subsequently, cells were continuously cultured in complete medium containing etoposide (refreshed every 48 h) for 2 to 3 weeks, until cell viability was maintained greater than 80%. Then etoposides were gradually added to medium to adjust concentrations to 3.75, 5, 7.5, and 10 μg/mL, respectively. At each concentration, the cultured cells grew stably and eventually attained 80% survival. The cells in stable logarithmic growth phase were cryopreserved. The cells that had been cultured with the highest concentration of etoposide were transferred to drug-free medium and cultured for 1 week as a drug-resistant strain for subsequent experiments. Resistance index = resistant strain IC_50_/parental strain IC_50_.

### Reverse Transcription–Quantitative Polymerase Chain Reaction

Total RNA was extracted from tissues or cells using TRIzol reagent (Invitrogen) and reversely transcribed into complementary DNA with Reverse Transcriptase MMLV (Promega Corporation, Madison, WI, United States), with RNA concentration measured by a NanoDrop 2000 microultraviolet spectrophotometer (011U; NanoDrop, Wilmington, DE, United States). The reverse transcription–quantitative polymerase chain reaction (RT-qPCR) was performed using CFX96 real-time fluorescence qPCR detection system (Bio-Rad, Hercules, CA, United States). Transcriptional level of genes was calculated by 2^−ΔΔCt^ quantification method with glyceraldehyde-3-phosphate dehydrogenase as internal reference. The primer sequences are shown in Supplementary Table S2.

### Western Blot Analysis

Protein was isolated in radioimmunoprecipitation assay (RIPA) lysis buffer (Beyotime Biotechnology, Beijing, China). Protein concentration was assayed using the bicinchoninic acid kit (Pierce, Rockford, IL, United States). Protein was then separated by 4% concentration gel and 10% polyacrylamide gel electrophoresis and then transferred to polyvinylidene fluoride membranes. The membrane was blocked with 5% skim milk powder for 1 h and incubated with the following primary antibodies at 4°C overnight: IGF2BP2 (11601-1-AP, 1:3,000, Proteintech, Wuhan, China), FOXO1 (ab52857, 1:2,000; Abcam Inc., Cambridge, United Kingdom), PID1 (27951, 1:1,000, SAB, United States), m6A (MABE1006, 1 μg/mL; Sigma–Aldrich), ubiquitin (ab7780, 1:2,000; Abcam), and β-actin (ab8227, 1:2,000; Abcam). Then the membrane was probed with secondary antibody horseradish peroxidase–labeled goat anti–rabbit/mouse (SA00001-1/SA00001-12, 1:5,000; Proteintech), followed by washing in phosphate-buffered saline (PBS) containing 0.1% Tween-20 six times for 5 min each. The membranes were developed using enhanced chemiluminescence (Thermo Fisher Scientific Inc., Waltham, MA, United States) and observed by a Bio-Rad ChemiDoc™ imaging system. The Western blot image was analyzed with ImageJ2x software. Relative expression of target protein was calculated as the ratio of the gray value of the target protein band to that of the internal reference β-actin band of the same sample.

### RNA Pull-Down Assay

RNA pull-down experiments were performed using the RNA-Protein Pull-Down Kit (Thermo Fisher Scientific) according to the manufacturer’s instructions. In brief, T4 RNA polymerase (Roche, Basel, Switzerland) was used to label DANCR, and biotinylated RNA was mixed with streptavidin magnetic beads (Invitrogen) at 4°C overnight, and then the cell lysate was placed on ice for 1 h. Finally, the protein level of FOXO1 in the RNA protein binding mixture was detected by Western blot analysis.

### RNA Binding Protein Immunoprecipitation Assay

IGF2BP2 or FOXO1 antibody was added to pull down DANCR with IgG (1:500, ab109489; Abcam) serving as NC, and protein A/G magnetic beads then recovered IGF2BP2 or FOXO1 antibody. RT-qPCR was conducted to confirm the presence of DANCR in the precipitate. Then, m6A antibody (MABE1006; Sigma) was added to pull down m6A (N6-methyladenine)–modified DANCR.

Total RNA was isolated from U251 cells in a 10-cm Petri dish and dissolved in 50 μL of RNase-free solution, with 5 μL as RNA input. The remaining 45 μL was added to 500 μL of RNase inhibitor–containing pull-down lysis buffer. The RNA was incubated with 1 μL of mouse IgG, and the IgG was recovered using protein A/G magnetic beads. The lysate was transferred to a new tube and mixed with IgG or m6A antibody and rotated overnight at 4°C along with protein A/G magnetic beads. Finally, TRIzol reagent was used to extract m6A-bound RNA, and the RNA level of DANCR was detected by RT-qPCR.

### *In Vivo*–Modified Streptavidin-Binding RNA Aptamer Precipitation

For the pull-down experiment of DANCR labeled by S1m (Leppek and Stoecklin, 2014) *in vivo*, the S1m vector or S1m-conjugated DANCR vector was transfected into U251 cells. After 48 h, the cells were collected and lysed in 1 mL lysis buffer containing proteinase inhibitors and RNase inhibitors. The mixture was centrifuged at 1,000*g* for 20 min at 4°C, and the supernatant was incubated with streptavidin magnetic beads for 30 min. Next, with 1/10 of the lysate as protein input, the other lysate was mixed with streptavidin magnetic beads at 4°C for 4 h. Finally, the magnetic bead pellet was washed with PBS and dissolved in 30 μL of 2× sodium dodecyl sulfate sample buffer, after which Western blot analysis was performed with IGF2BP2 antibody.

S1m-DANCR was pulled down as above and then washed with RIP buffer three times. The magnetic beads pellet was resuspended in 1-mL pull-down buffer and incubated with m6A antibody (2 µl) overnight. S1m-DANCR-m6A antibody complex was detected by Western blot analysis.

### Ubiquitination Assay

Ubiquitin plasmids (Ub, Ubbiotech, China) were cotransfected into U251 cells with pHAGE-DANCR and pHAGE-FOXO1 plasmids. After 36 h, MG132 was added to the medium and incubated for 8 h. Thereafter, the cells were collected and subjected to immunoprecipitation with FOXO1 antibody, whereas Ub antibody was added to detect the level of ubiquitination by Western blot analysis.

### Dual-Luciferase Reporter Gene Assay

The binding region “TATTTT” of FOXO1 at transcription start site of PID1 or mutant (MUT) sequence “ATAAAA” was cloned upstream of the pGL4 reporter vector luciferase gene. pHAGE-NC (empty plasmids) or pHAGE-FOXO1 plasmids were cotransfected into U251 cells with the reporter plasmids pGL4-PID1 reporter or pGL4-PID1-MUT reporter and pRL-TK (with the grouping of pHAGE-NC + pGL4-PID1 reporter + pRL-TK, pHAGE-NC + pGL4-PID1-MUT reporter + pRL-TK, pHAGE-FOXO1 + pGL4-PID1 reporter + pRL-TK and pHAGE-FOXO1 + pGL4-PID1-MUT reporter + pRL-TK). After 48 h, the luciferase activity was determined using the Bio Tek Synergy 2 dual-luciferase reporter gene detection system. The relative luciferase activity of target reporter genes was calculated as the ratio of luciferase activity of firefly luciferase to that of Renilla luciferase.

### MTT Assay

Cell suspension was seeded into a 96-well plate and incubated at 37°C in 5% CO_2_. After 24 h, cells were treated with different concentrations of etoposide for 48 h. After removal of the medium, 20 μL of MTT solution (5 mg/mL, 0.5% MTT) and 90 μL of serum-free DMEM were added to each well and cultured for 4 h, whereupon the supernatant was removed. Next, 200 μL dimethyl sulfoxide was added, and the sample was rotated for 10 min to dissolve crystals. Optical density value at 570 nm (OD_570_) was measured by an automatic microplate reader, and viability rate was calculated as follows: cell viability (%) = OD_570_ of experimental group/OD_570_ of control group × 100%, presented in a curve.

### Colony Formation Assay

Cells were seeded on six-well culture plates at a density of 1 × 10^3^ cells/well and placed in an incubator (37°C, 5% CO_2_) for 10–15 days. The medium was renewed every 4–5 days until colonies were visible in the Petri dish. The supernatant was then discarded, and the cells were washed twice with PBS. Colonies were fixed with 5 mL 4% paraformaldehyde and stained with 0.5% crystal violet for 10–30 min. The number of cell colonies was observed in three randomly selected fields of view, and the assay was independently conducted for three times. The colony formation rate = number of colonies/number of seeded cells.

### Flow Cytometry

GBM cells were seeded on six-well plates at a density of 2 × 10^5^ cells/well and subjected to lentivirus-mediated transduction as described above. Then, 48 h after the transduction, the cells were washed with precooled PBS and trypsinized. The cells were centrifuged at 800*g*, with the supernatant removed. After two washes in PBS, the precipitate was determined by annexin V–fluorescein isothiocyanate (FITC) apoptosis kit, whereas cells were resuspended in 500 μL binding solution and then mixed with 5 μL FITC and 5 μL propidium iodide for 15 min. After rotation, cell apoptosis rate was analyzed with a flow cytometer (BD FACSCalibur, Franklin Lakes, NJ, United States).

### Immunohistochemistry

Tumor tissues were collected from mice of each group (n = 8) for immunohistochemical detection. The tumor tissue sections were immersed in 3% hydrogen peroxide for blocking of endogenous peroxidase. The sections were then heated in 10 mM sodium citrate and blocked in 10% normal goat serum. Thereafter, the sections were incubated with anti-Ki67 antibody (ab15580, 1:1,000; Abcam) overnight in a wet room at 4°C. The next day, the sections were incubated with secondary antibody for 1 h at room temperature. The DAB kit was used for immunoreactivity detection. Three fields of view were randomly selected in each sample for microscopic observation, and images were photographed. The final degree of immune response was determined by the sum of staining intensity and positive staining area (Zhao et al., 2015).

### Mouse Xenograft Model

BALB/c-nu female nude mice obtained from Beijing Vital River Laboratory Animal Technology Co., Ltd., were maintained under specific pathogen free condition. U251 cells were transduced with lentiviral vectors carrying pSUPER-sh-NC + vector, pSUPER-sh-IGF2BP2 + vector, pSUPER-sh-NC + pHAGE-DANCR, pSUPER-sh-IGF2BP2 + pHAGE-DANCR; vector, pHAGE-IGF2BP2, pHAGE-PID1, pHAGE-IGF2BP2 + pHAGE-PID1, or pHAGE-DANCR + pHAGE-PID1. Thereafter, glioma cells in the logarithmic growth phase were digested and prepared with serum-free 1640 medium to a single cell suspension (2 × 10^7^ cells/mL). The single cell suspension was inoculated (0.1 mL/mouse) under the skin of the adjacent part of the left hind leg and abdomen of nude mice, with eight mice in each group. Etoposide was intragastrically administered daily over 6 days in 0.5% methylcellulose at a dose of 80 mg/kg per day, and control mice received vehicle (0.5% methylcellulose) (Panigrahy et al., 2010). Meanwhile, another group of tumor-bearing mice (n = 8) was subjected to survival analysis.

An *in vivo* imaging system (IVIS Lumina; Perkin Elmer, Norwalk, CT, United States) was used to track the growth of tumors formed by luciferase-labeled cancer cells, and the xenografted tumor in nude mice was evaluated according to the *in vivo* intensity of luciferase. Then, the mice and inoculation site were evaluated every day, and the survival of nude mice was observed. Nude mice were euthanized by intraperitoneal injection of 80 mg/kg pentobarbital sodium, and the tumor tissue was isolated. The expression of Ki67 protein in the tumor tissue was detected by immunohistochemistry.

### Microarray-Based Gene Expression Profiling

Differentially expressed genes in glioma samples were screened from The Cancer Genome Atlas (TCGA) database using the Gene Expression Profiling Interactive Analysis (GEPIA) tool with |Log_2_FC| >2 and *p* < 0.01 as the threshold and one-way analysis of variance (ANOVA). In order to predict the downstream regulatory factors of IGF2BP2 in glioma, GEPIA was used to analyze the correlation between genes and Pearson correlation coefficient to evaluate the correlation of genes in glioma. Gene interaction factors were predicted using the BioGRID website, and the target gene of the regulatory factor was predicted by the hTFtarget website. Multi Experiment Matrix (MEM) was adopted to determine the related gene of the regulatory factor and to rank other genes based on the similarity in each individual dataset. Glioma-related genes were retrieved from the GeneCards database and then subjected to intersection analysis using the jvenn tool. The coexpression relationship between genes was analyzed using the Coexpedia website.

### Statistical Analysis

Data were processed using GraphPad Prism 5 software. Values shown represent mean ± standard deviation of three independently performed experiments. Data between two groups were performed by unpaired *t* test. Multigroup data comparison was conducted by one-way ANOVA followed by Tukey *post hoc* test. Data among groups with different concentration or at different time points were compared by two-way ANOVA, followed by Tukey *post hoc* test. Pearson correlation coefficient was used to analyze the correlation of indicators, and nude mouse survival was analyzed by Kaplan–Meier method with log-rank test univariate analysis. *p* < 0.05 was considered statistically significant.

## Results

### IGF2BP2 Is Upregulated in Glioma

**FIGURE 1:**
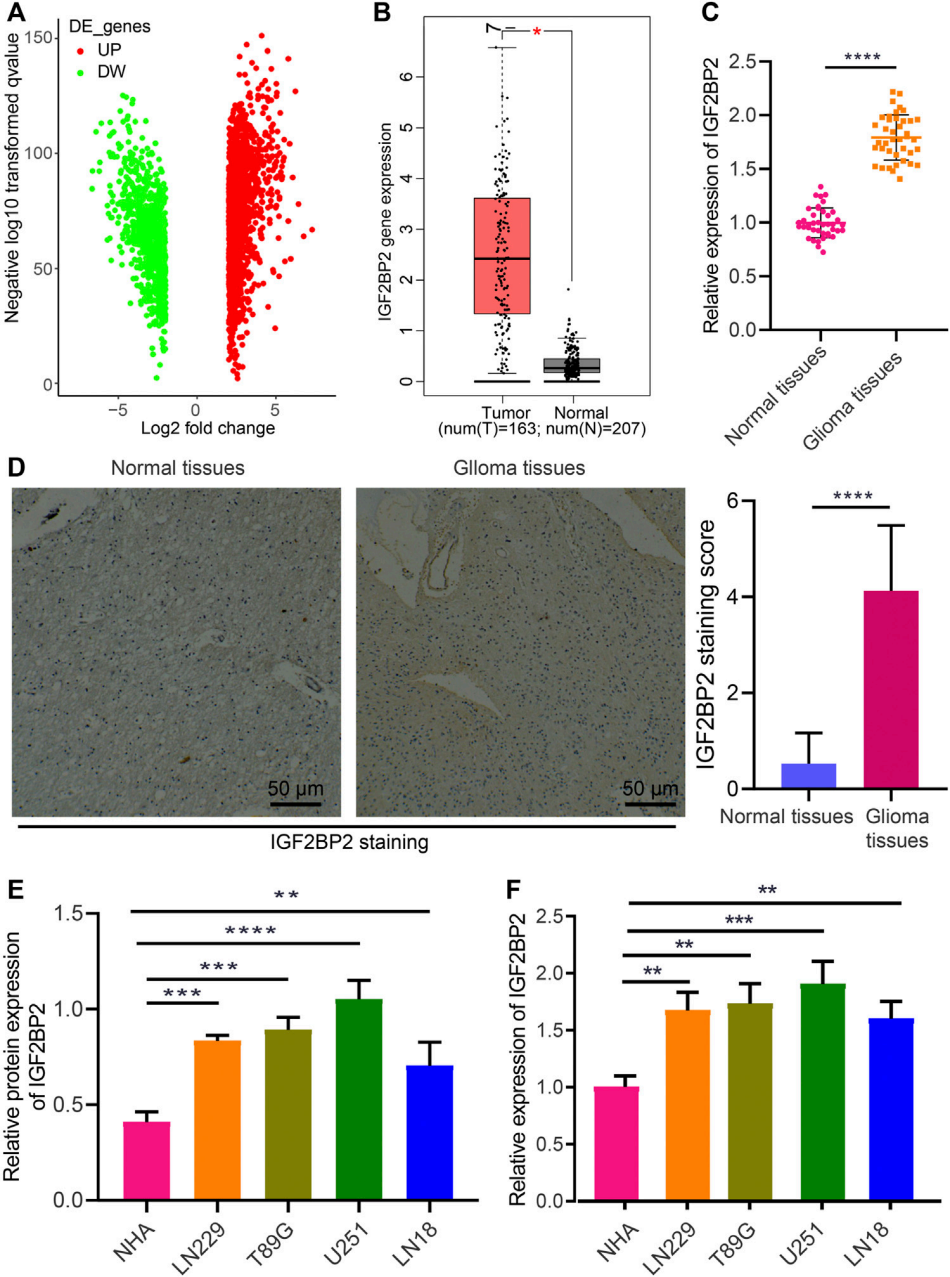
Aberrant expression of IGF2BP2 in glioma cells and tissues. **(A)** Volcano plot of differentially expressed genes in glioma samples and normal samples in the TCGA database. DE, differentially expressed genes; UP, upregulated; DW, downregulated. **(B)** Expression of IGF2BP2 in glioma samples in the TCGA database. **(C)** RT-qPCR analysis of IGF2BP2 mRNA expression in glioma tissues and normal brain tissues. **(D)** Immunohistochemistry of IGF2BP2 protein in glioma tissues and normal brain tissues. **(E)** Western blot analysis of IGF2BP2 protein in GBM cell lines (U251MG, LN229, LN18, T98G) and the primary normal human astrocyte NHA cell line. **(F)** RT-qPCR analysis of IGF2BP2 mRNA expression in GBM cell lines (U251MG, LN229, LN18, T98G) and the primary normal human astrocyte NHA cell line. *p < 0.05, **p < 0.01, ***p < 0.001, ****p < 0.0001. Values shown represent mean ± standard deviation of three independently performed experiments. Data between two groups were compared by unpaired *t* test. Multigroup data comparison were conducted by one-way ANOVA followed by Tukey *post hoc* test.

To understand the role of IGF2BP2 in glioma, differential expression analysis was performed based on glioma and normal samples in the TCGA database, which identified a total of 2,281 differentially expressed genes (Figure 1A). Among these genes, IGF2BP2 was significantly highly expressed in glioma (Figure 1B). To confirm IGF2BP2 expression in glioma, we used RT-qPCR to determine the mRNA expression of IGF2BP2 in human glioma tissues and normal brain tissues. In line with bioinformatics analysis, IGF2BP2 was observed to be upregulated in glioma tissue relative to normal brain tissues (Figure 1C). Similarly, the immunohistochemistry confirmed the upregulated expression of IGF2BP2 in glioma tissues (Figure 1D). Furthermore, we checked the expression of IGF2BP2 in various GBM cell lines (U251, LN229, LN18, and T98G), all of which presented with higher IGF2BP2 expression level relative to primary normal human astrocytes (NHA) (Figures 1E,F). Collectively, these data indicated that IGF2BP2 was highly expressed in glioma.

### IGF2BP2 Elevates DANCR Expression and Enhances Stability of DANCR

**FIGURE 2:**
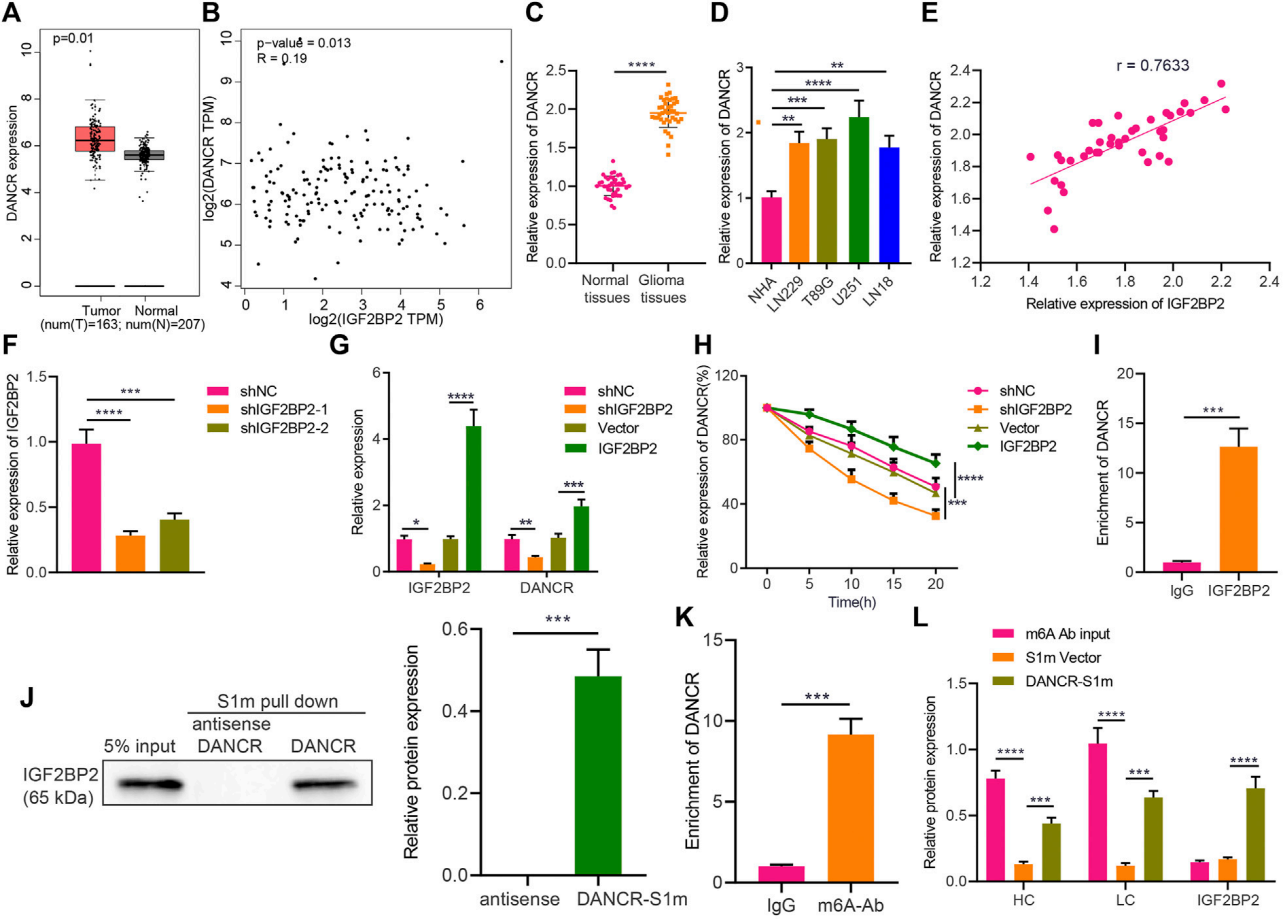
IGF2BP2 enhances stability of DANCR in GBM cells. **(A)** GEPIA analysis of DANCR expression in glioma samples. **(B)** Correlation between IGF2BP2 and DANCR genes in TCGA database-based glioma samples (n = 163) analyzed by GEPIA. **(C)** RT-qPCR analysis of DANCR mRNA expression in glioma tissues and normal brain tissues. **(D)** RT-qPCR analysis of DANCR mRNA expression in GBM cell lines (U251MG, LN229, LN18, T98G) and the primary normal human astrocyte NHA cell line. **(E)** Pearson correlation coefficient analysis between IGF2BP2 and DANCR in glioma tissues. **(F)** RT-qPCR analysis of efficiency of IGF2BP2 silencing in U251 cells upon treatment with sh-IGF2BP2-1 or sh-IGF2BP2-2. **(G)** RT-qPCR analysis of DANCR mRNA expression in U251 cells upon treatment with sh-IGF2BP2, vector, sh-NC, or IGF2BP2. **(H)** RT-qPCR analysis of DANCR mRNA expression in U251 cells upon treatment with sh-IGF2BP2, vector, sh-NC, or IGF2BP2 following actinomycin D treatment. **(I)** RIP assay of DANCR enrichment with IGF2BP2 antibody or IgG in U251 cells. **(J)** Western blot analysis of IGF2BP2 in the DANCR complex pulled down by streptavidin magnetic beads in U251 cells transfected with pHAGE-S1m-DANCR plasmids. Input refers to the whole cell lysate. **(K)** RIP assay of DANCR enrichment with m6A antibody or IgG in U251 cells. **(L)** Western blot analysis of IGF2BP2 expression in the pull-down complex of U251 cells treated with DANCR-S1m, m6A antibody, input, or S1m vector. HL indicates heavy chain and LC indicates light chain. *p < 0.05, **p < 0.01, ***p < 0.001, ****p < 0.0001. Values shown represent mean ± standard deviation of three independently performed experiments. Data between two groups were compared by unpaired *t* test. Multigroup data comparison was conducted by one-way ANOVA followed by Tukey *post hoc* test. Data among groups at different time points were compared by two-way ANOVA, followed by Tukey *post hoc* test.

A recent study has highlighted that IGF2BP2 may serve as a reader for m6A-modified DANCR and stabilize DANCR RNA in pancreatic cancer (Hu et al., 2020). To identify the potential relationship between IGF2BP2 and DANCR in glioma, we conducted a correlation analysis through GEPIA, which indicated a significant correlation between them, whereas DANCR was also identified to be highly expressed in glioma (Figures 2A,B). We then confirmed the upregulated expression of DANCR both in glioma tissues and various GBM cells (U251MG, LN229, LN18, and T98G) (Figures 2C,D). Pearson correlation coefficient in glioma tissues identified a positive correlation between IGF2BP2 and DANCR expression (Figure 2E).

Furthermore, the results of RT-qPCR confirmed that IGF2BP2 expression was downregulated in U251 cells treated with sh-IGF2BP2-1 or sh-IGF2BP2-2, of which sh-IGF2BP2-1 exhibited better silencing efficiency (Figure 2F) and was therefore selected for the subsequent experiments. In addition, U251 cells treated with sh-IGF2BP2 exhibited a remarkable decrease in DANCR expression, whereas an opposite result was noted in U251 cells overexpressing IGF2BP2 (Figure 2G). These data suggest that IGF2BP2 might upregulate DANCR expression in GBM cells.

Furthermore, to understand the role of IGF2BP2 in regulating DANCR, we constructed IGF2BP2 overexpression/silencing U251 cells and treated them with 2 μg/mL of actinomycin D, an RNA synthesis inhibitor. It was evident that silencing IGF2BP2 facilitated the degradation of DANCR, whereas overexpression of IGF2BP2 slowed down the rate of its degradation (Figure 2H), indicating that IGF2BP2 can promote DANCR stability. To assess the binding between IGF2BP2 and DANCR, we conducted RIP in U251 cells, with the results showing that IGF2BP2 antibody could pull down more DANCR than IgG antibody (Figure 2I), thus confirming the binding of IGF2BP2 to DANCR. Then, U251 cells were treated with the S1 streptavidin-labeled DANCR plasmid or the antisense strand (serving as NC), and the cell extracts were pulled down with streptavidin magnetic beads. Western blot analysis revealed that IGF2BP2 was detected in the DANCR-S1m pull-down complex and not in the antisense strand (Figure 2J).

m6A is the most prevalent modification in eukaryotic mRNAs, and members of the IGF2BP family can promote its recognition and the stability of target RNA, dependent on IGF2BP (Huang et al., 2018). According to RIP assay using m6A antibody to pull down DANCR, compared with the control IgG treatment, addition of m6A antibody resulted in enrichment of DANCR in GBM cells (Figure 2K), suggesting that DANCR in GBM cells was indeed modified by methylation. To further investigate the methylation, we carried out a special interactive RNA precipitation where the S1 streptavidin-labeled DANCR plasmid or control plasmid carried by lentivirus was transduced into U251 cells, and DANCR-S1m was then pulled down by streptavidin magnetic beads, followed by Western blot analysis (Figure 2L). Heavy and light chains were detected in the DANCR-S1m pull-down sample, validating that DANCR can be modified by methylation in GBM cells. In conclusion, in GBM cells, DANCR can be modified by methylation, and IGF2BP2 can recognize methylated DANCR and promote its stability.

### IGF2BP2 Promotes the Etoposide Resistance Through DANCR in GBM Cells

**FIGURE 3:**
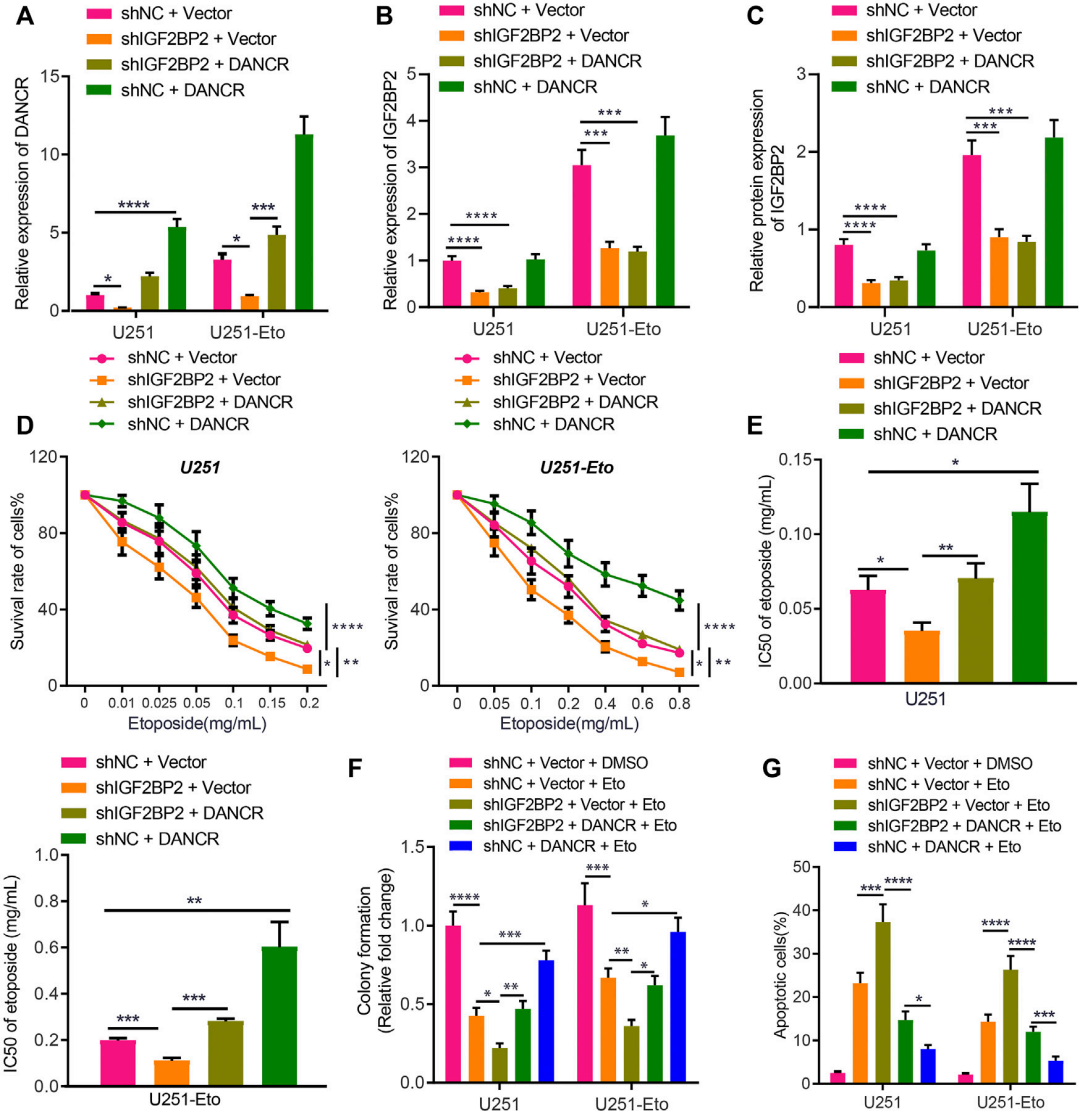
IGF2BP2 enhances DANCR stability and induces etoposide resistance of GBM cells. **(A)**. RT-qPCR analysis of DANCR mRNA expression in U251 and U251-Eto cells upon treatment with sh-NC + vector, sh-IGF2BP2 + vector, sh-IGF2BP2 + DANCR, or sh-NC + DANCR. **(B)** RT-qPCR analysis of IGF2BP2 mRNA expression in U251 and U251-Eto cells upon treatment with sh-NC + vector, sh-IGF2BP2 + vector, sh-IGF2BP2 + DANCR, or sh-NC + DANCR. **(C)** Western blot analysis of IGF2BP2 protein in U251 and U251-Eto cells upon treatment with sh-NC + vector, sh-IGF2BP2 + vector, sh-IGF2BP2 + DANCR, or sh-NC + DANCR. **(D)** MTT analysis of U251 and U251-Eto cell viability upon treatment with sh-NC + vector, sh-IGF2BP2 + vector, sh-IGF2BP2 + DANCR, or sh-NC + DANCR. **(E)** IC_50_ of etoposide in U251 and U251-Eto cells upon treatment with sh-NC + vector, sh-IGF2BP2 + vector, sh-IGF2BP2 + DANCR, or sh-NC + DANCR. **(F)** Quantification of colony formation assay on U251 and U251-Eto cells upon treatment with sh-NC + vector, sh-IGF2BP2 + vector, sh-IGF2BP2 + DANCR, or sh-NC + DANCR. **(G)** Flow cytometric analysis of U251 and U251-Eto cell apoptosis upon treatment with sh-NC + vector, sh-IGF2BP2 + vector, sh-IGF2BP2 + DANCR, or sh-NC + DANCR. *p < 0.05, **p < 0.01, ***p < 0.001, ****p < 0.0001. Values shown represent mean ± standard deviation of three independently performed experiments. Multigroup data comparison was conducted by one-way ANOVA followed by Tukey *post hoc* test. Data among groups at different concentrations were compared by two-way ANOVA, followed by Tukey *post hoc* test.

Following the aforementioned findings, we then explored the potential role of IGF2BP2 stabilizing DANCR in etoposide resistance of GBM cells by culturing and screening out etoposide-resistant U251-Eto strain cells. As shown by results of the MTT assay, the IC_50_ of the parent U251 was 0.06 ± 0.01 mg/mL, and that of U251-Eto was 0.2 ± 0.01 mg/mL (Figures 3D,E). Subsequent related experiments were carried out with the corresponding IC_50_ concentrations. According to RT-qPCR and Western blot analysis, sh-IGF2BP2 decreased not only IGF2BP2 expression but also lowered the DANCR level. In response to sh-IGF2BP2 + vector and sh-IGF2BP2 + DANCR treatment, IGF2BP2 expression was downregulated, whereas sh-IGF2BP2 + DANCR and sh-NC + DANCR led to elevated expression of DANCR. Importantly, compared to parent U251 cells, U251-Eto cells exhibited a higher level of DANCR and IGF2BP2 (Figures 3A–C).

To identify the impact of DANCR and IGF2BP2 on drug resistance, GBM U251 cells were exposed to different concentration of etoposide, followed by MTT assay. We found that IGF2BP2 knockdown induced lower viability of GBM U251 and U251-Eto cells with decreased IC_50_, but overexpression of DANCR increased the viability and IC_50_ significantly, reversing the impact of IGF2BP2 knockdown (Figures 3D,E). Besides, colony formation assay and flow cytometry demonstrated that silencing IGF2BP2 potentiated the effect of etoposide to promote apoptosis and inhibit survival of glioma cells, whereas overexpression of DANCR exerted the opposite effect, abrogating the effect of sh-IGF2BP2 on glioma cells (Figures 3F,G; Supplementary Figure S1A). These data elucidated that IGF2BP2 induced etoposide resistance of GBM cells by promoting DANCR expression. We then aimed to understand the *in vivo* roles of IGF2BP2 and DANCR in etoposide resistance. It was found that sh-IGF2BP2 decreased bioluminescence, diminished Ki67 protein expression, and increased survival of mice. DANCR overexpression, in contrast, elevated bioluminescence, increased Ki67 protein expression, and deceased mouse survival (Supplementary Figures S2A–C; Supplementary Table S3). These *in vivo* data supported the findings of *in vitro* experiments that IGF2BP2 induced the resistance to etoposide in GBM cells by promoting DANCR expression.

### DANCR Targets FOXO1 and Negatively Regulates Its Expression in Glioma

**FIGURE 4:**
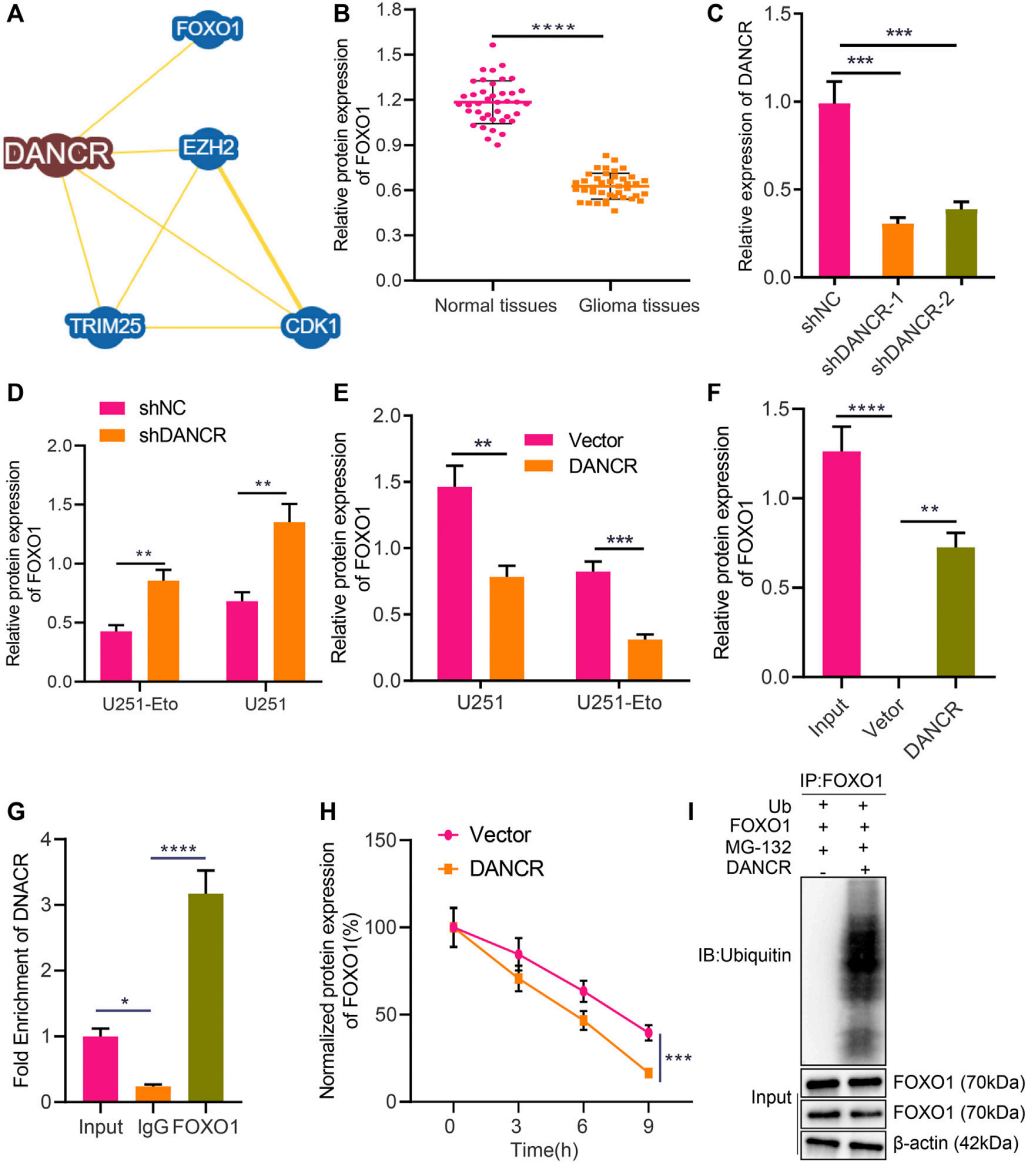
DANCR promotes FOXO1 ubiquitination and degradation in glioma cells. **(A)** Interaction factors of DANCR predicted by the BioGRID website. **(B)** Western blot analysis of FOXO1 protein in glioma tissues and normal brain tissues. **(C)** RT-qPCR analysis of efficiency of DANCR silencing in U251 cells upon treatment with sh-DANCR-1 or sh-DANCR-2. **(D)** RT-qPCR analysis of FOXO1 mRNA expression in U251 cells and U251-Eto upon treatment with sh-NC, or sh-DANCR. **(E)** Western blot analysis of FOXO1 protein in U251 cells and U251-Eto upon treatment with sh-NC, or sh-DANCR. **(F)** Binding between FOXO1 and DANCR analyzed by RNA pull-down assay in U251 cells. **(G)** Binding between FOXO1 and DANCR confirmed by RIP assay. **(H)** Western blot analysis of FOXO1 protein upon treatment with CHX and transduction with DANCR or vector at 0, 3, 6, 9 h after administration. **(I)** Ubiquitination experiment of FOXO1 protein ubiquitination upon treatment with Ub, DANCR, or FOXO1. *p < 0.05, **p < 0.01, ***p < 0.001, ****p < 0.0001. Values shown represent mean ± standard deviation of three independently performed experiments. The unpaired *t* test was used for the comparison between data of two groups. Multigroup data comparison was conducted by one-way ANOVA followed by Tukey *post hoc* test. Data among groups at different time point were compared by two-way ANOVA, followed by Tukey *post hoc* test.

Recent literature has revealed that FOXO1 enhances etoposide-induced cytotoxicity against glioma cells (Ni et al., 2019). In this study, we were interested in identifying the role of FOXO1 in glioma and its association with FOXO1. The BioGRID website identified FOXO1 as an interaction factor of DANCR (Figure 4A). For further verification, we displayed that FOXO1 was poorly expressed in glioma tissues relative to normal brain tissues (Figure 4B). We established sh-DANCR-1 and sh-DANCR-2 sequences, whose knockdown efficiency was confirmed by RT-qPCR. The results presented that sh-DANCR-1 exhibited superior knockdown efficiency (Figure 4C), and it was thus selected for subsequent experiments. In addition, in U251 cells or U251-Eto cells overexpressing DANCR, FOXO1 was poorly expressed; conversely, silencing of DANCR increased the expression of FOXO1 (Figures 4D,E). Besides, the results of RNA pull-down and RIP assays demonstrated that DANCR pulled down FOXO1 and that DANCR enrichment by FOXO1 was elevated relative to that by IgG or Input (Figures 4F,G), indicating the occurrence of binding between DANCR and FOXO1 in glioma cells. Moreover, silencing of DANCR prolonged the half-life of FOXO1 expression (Figure 4H), which suggested that the binding of DANCR to FOXO1 can weaken the stability of FOXO1 protein. Ubiquitination experimental results further elucidated that DANCR elevated the ubiquitination level of FOXO1 (Figure 4I). Taken together, these results indicated that DANCR interacted with FOXO1 to promote the ubiquitination of FOXO1, thereby inhibiting the protein expression of FOXO1.

### DANCR Induces Etoposide Resistance of GBM Cells by Inhibiting FOXO1 Expression

**FIGURE 5:**
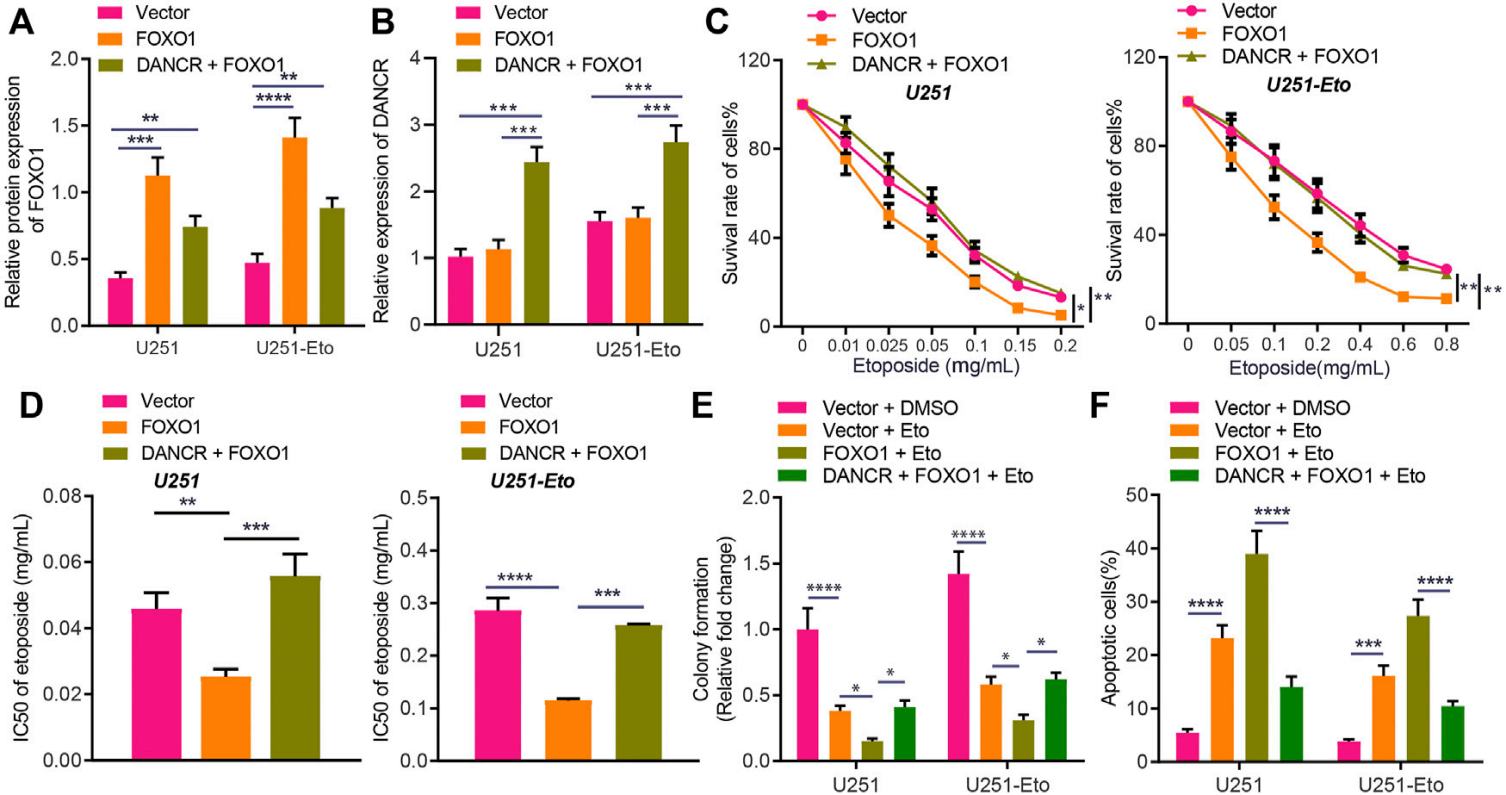
DANCR enhances etoposide resistance of GBM cells by inhibiting FOXO1 expression. **(A)** Western blot analysis of FOXO1 protein in U251 and U251-Eto cells treated with FOXO1, DANCR, FOXO1 + DANCR, or vector. **(B)** RT-qPCR analysis of DANCR mRNA expression in U251 and U251-Eto cells treated with FOXO1, DANCR, FOXO1 + DANCR, or vector. **(C)** MTT analysis of U251 and U251-Eto cell viability upon treatment with FOXO1, DANCR, FOXO1 + DANCR, or vector. **(D)** IC_50_ of etoposide in U251 and U251-Eto cells upon treatment with FOXO1, DANCR, FOXO1 + DANCR, or vector. **(E)** Quantification of colony formation assay on U251 and U251-Eto cells upon treatment with FOXO1, DANCR, FOXO1 + DANCR, or vector. **(F)** Flow cytometric analysis of U251 and U251-Eto cell apoptosis upon treatment with FOXO1, DANCR, FOXO1 + DANCR, or vector. *p < 0.05, **p < 0.01, ***p < 0.001, ****p < 0.0001. Values shown represent mean ± standard deviation of three independently performed experiments. Multigroup data comparison was conducted by one-way ANOVA followed by Tukey *post hoc* test. Data among groups at different concentrations were compared by two-way ANOVA, followed by Tukey *post hoc* test.

To further investigate whether DANCR inhibits FOXO1 and thereby promotes etoposide resistance of GBM cells, we cultured U251 and U251-Eto cell lines and constructed cells overexpressing of DANCR or FOXO1. FOXO1 was observed to be downregulated in U251-Eto cells relative to parental U251 cells. The results of Western blot analysis and RT-qPCR confirmed that DANCR and FOXO1 expression was upregulated in U251 cells overexpressing DANCR and FOXO1, respectively, whereas overexpression of DANCR led to a reduction in FOXO1 expression (Figures 5A,B). MTT assay data showed that overexpression of FOXO1 decreased survival of GBM U251 and U251-Eto cells, accompanied with decreased content of IC_50_, whereas additional treatment with overexpression of DANCR restored the effect induced by FOXO1 (Figures 5C,D). Besides, the results of colony formation assay and flow cytometry revealed that GBM cells overexpressing FOXO1 had increased apoptosis and decreased survival, but overexpression of DANCR could negate this trend (Figures 5E,F). Collectively, our data unraveled that DANCR promoted the resistance of GBM cells to etoposide through inhibition of FOXO1.

### FOXO1 Decreases the Etoposide Resistance of GBM Cells Through PID1

**FIGURE 6:**
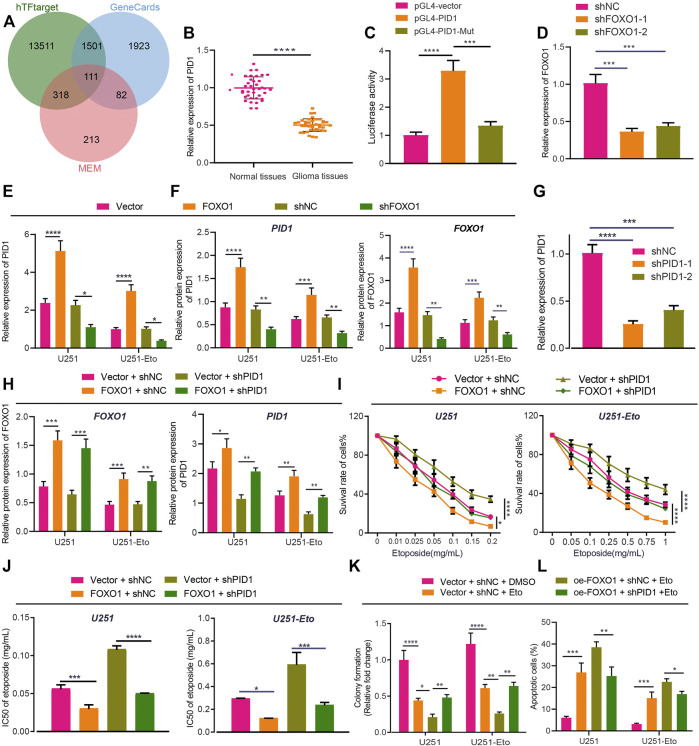
FOXO1 increases sensitivity of GBM cells to etoposide chemotherapy through PID1 promotion. **(A)** Venn diagram of FOXO1 related genes, target genes and glioma genes screened from the hTFtarget website, MEM and GeneCards dataset. **(B)** RT-qPCR analysis of PID1 mRNA expression in clinical glioma tissues and normal brain tissues. **(C)** Luciferase activity of PID1 in U251 cells upon treatment with FOXO1 plasmids determined by dual-luciferase reporter gene assay. **(D)** RT-qPCR analysis of efficiency of PID1 silencing in U251 and U251-Eto cells upon treatment with sh-FOXO1-1 or sh-FOXO1-2. **(E)** RT-qPCR analysis of PID1 mRNA expression in U251 and U251-Eto cells upon treatment with FOXO1, sh-FOXO1, sh-NC, or vector. **(F)** Western blot analysis of PID1 and FOXO1 proteins in U251 and U251-Eto cells upon treatment with FOXO1, sh-FOXO1, sh-NC, or vector. **(G)** RT-qPCR analysis of efficiency of PID1 silencing in U251 and U251-Eto cells upon treatment with sh-PID1-1 or sh-PID1-2. **(H)** Western blot analysis of PID1 and FOXO1 proteins in U251 and U251-Eto cells upon treatment with vector + sh-NC, vector + sh-PID1, FOXO1 + sh-NC, or FOXO1 + sh-PID1. **(I)** MTT analysis of U251 and U251-Eto cell viability upon treatment with vector + sh-NC, vector + sh-PID1, FOXO1 + sh-NC, or FOXO1 + sh-PID1. **(J)** IC_50_ of etoposide in U251 and U251-Eto cells upon treatment with vector + sh-NC, vector + sh-PID1, FOXO1 + sh-NC, or FOXO1 + sh-PID1. **(K)** Quantification of colony formation assay on U251 and U251-Eto cells upon treatment with vector + sh-NC, vector + sh-PID1, FOXO1 + sh-NC, or FOXO1 + sh-PID1. **(L)** Flow cytometric analysis of U251 and U251-E to cell apoptosis upon treatment with FOXO1, DANCR, FOXO1 + DANCR, or vector. In panel A–G, *p < 0.05, **p < 0.01, ***p < 0.001, ****p < 0.0001. In panel H–L, *p < 0.05, **p < 0.01, ***p < 0.001, ****p < 0.0001. Values shown represent mean ± standard deviation of three independently performed experiments. Data between two groups were compared by unpaired *t* test. Multigroup data comparison was conducted by one-way ANOVA followed by Tukey *post hoc* test. Data among groups at different concentrations were compared by two-way ANOVA, followed by Tukey *post hoc* test.

To understand the mechanism underlying FOXO1 functioning in glioma, we continued to explore its target genes. A total of 15,441 target genes of FOXO1 were obtained from hTFtarget website, 724 FOXO1-related genes from MEM analysis, and 3,617 glioma-related genes from GeneCards database with 111 candidate genes at the intersection (Figure 6A). These 111 candidates were analyzed by Coexpedia website and displayed in a coexpression network, including PID1 (Supplementary Figure S3). Meanwhile, PID1 has been reported to promote the sensitivity of GBM cells to etoposide, and FOXO1 can bind to the promoter region of PID1 (Erdreich-Epstein et al., 2014; Xu et al., 2017; Zhao et al., 2017). In this study, we attempted to identify the interaction between FOXO1 and PID1 in glioma. Initially, RT-qPCR results indicated lower PID1 expression in clinical glioma tissues relative to normal brain tissues (Figure 6B). Dual-luciferase reporter gene assay demonstrated that FOXO1 promoted transcriptional activation of wild-type PID1 promoter rather than MUT PID1 promoter (Figure 6C). Either sh-FOXO1-1 or sh-FOXO1-2 inhibited mRNA and protein expression of PID1, with sh-FOXO1-1 selected for following experiments due to optimal silencing efficiency. Besides, overexpressing FOXO1 was associated with increased PID1 level (Figures 6D–F). In a word, FOXO1 bound to PID1 promoter, promoting transcription of PID1 in glioma.

To understand the role of FOXO1 binding to PID1 in GBM cell sensitivity to chemotherapy, we established sh-PID1-1 and sh-PID1-2 sequences, the efficiency of which was verified in GBM cells, with sh-PID1-1 selected for subsequent assays due to its superior silencing efficiency (Figure 6G). Moreover, the results of Western blot analysis presented that overexpression of FOXO1 increased the expression of PID1 protein in U251 and U251-Eto cells (Figure 6H). The treated cells then were administered with etoposide, followed by experiments to assess cell survival. Silencing of PID1 expression increased the viability and survival of GBM U251 cells and decreased apoptosis, which could be restored by addition of overexpressed FOXO1 (Figures 6I–L; Supplementary Figure S1B). Altogether, FOXO1 promoted PID1 expression and thus sensitized GBM cells to etoposide.

### IGF2BP2 Induces Etoposide Resistance in GBM Cells Through DANCR/PID1 Axis *In Vitro* and *In Vivo*

**FIGURE 7:**
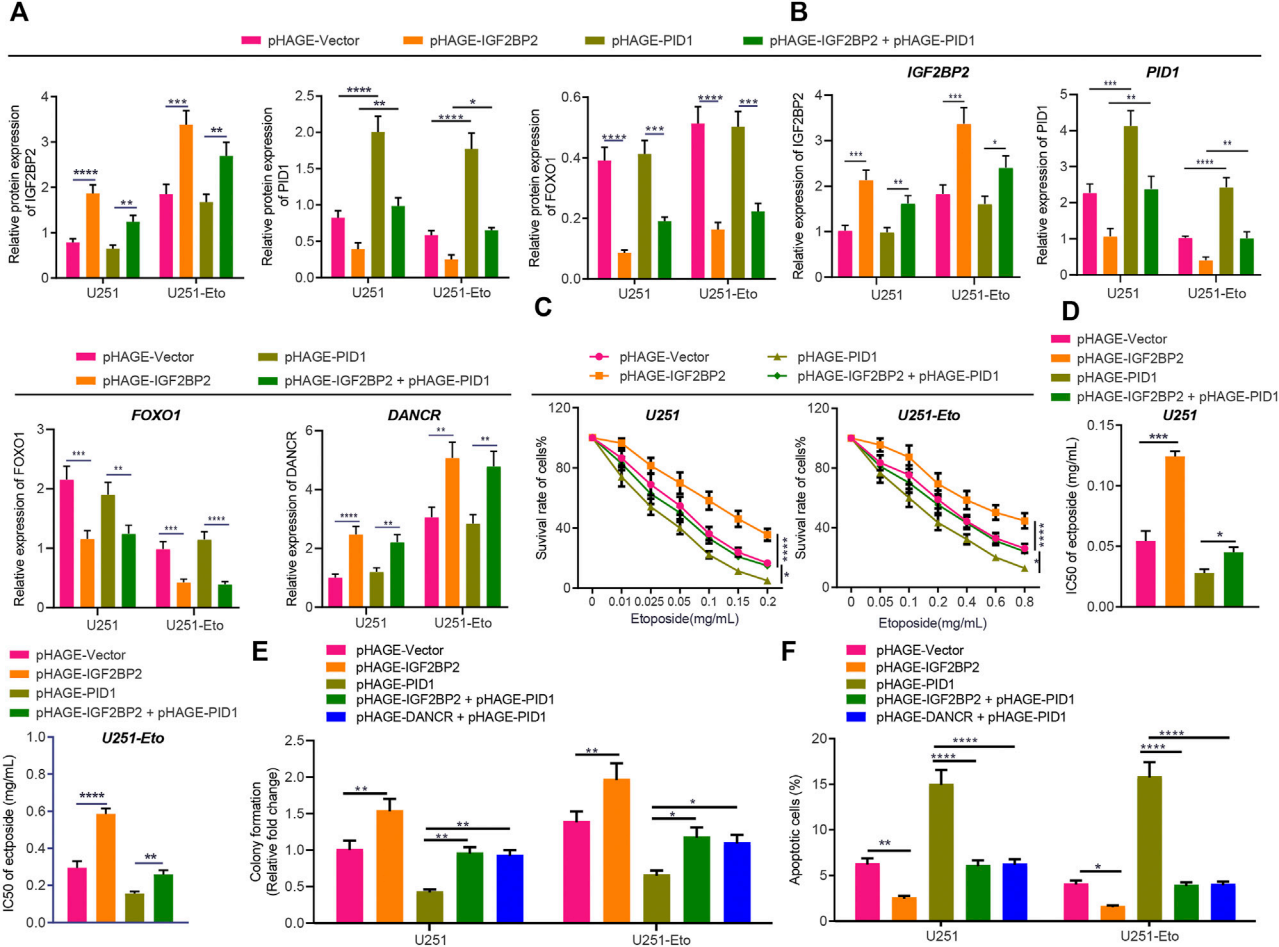
IGF2BP2 decreases sensitivity of GBM cells through the DANCR/PID1 axis. **(A)** Western blot analysis of FOXO1 protein in U251 and U251-Eto cells treated with pHAGE-vector, pHAGE-IGF2BP2, pHAGE-PID1, or pHAGE-IGF2BP2 + pHAGE-PID1. **(B)** RT-qPCR analysis of DANCR mRNA expression in U251 and U251-Eto cells treated with pHAGE-vector, pHAGE-IGF2BP2, pHAGE-PID1, or pHAGE-IGF2BP2 + pHAGE-PID1. **(C)** MTT analysis of U251 and U251-Eto cell viability upon treatment with pHAGE-vector, pHAGE-IGF2BP2, pHAGE-PID1, or pHAGE-IGF2BP2 + pHAGE-PID1. **(D)** IC_50_ of etoposide in U251 and U251-Eto cells upon treatment with pHAGE-vector, pHAGE-IGF2BP2, pHAGE-PID1, or pHAGE-IGF2BP2 + pHAGE-PID1. **(E)** Quantification of colony formation assay on U251 and U251-Eto cells upon treatment with pHAGE-vector, pHAGE-IGF2BP2, pHAGE-PID1, pHAGE-IGF2BP2 + pHAGE-PID1, or pHAGE-DANCR + pHAGE-PID1. **(F)** Flow cytometric analysis of apoptosis of U251 and U251-Eto cells upon treatment with pHAGE-vector, pHAGE-IGF2BP2, pHAGE-PID1, pHAGE-IGF2BP2 + pHAGE-PID1, or pHAGE-DANCR + pHAGE-PID1. *p < 0.05, **p < 0.01, ***p < 0.001, ****p < 0.0001. Values shown represent mean ± standard deviation of three independently performed experiments. Multigroup data comparison was conducted by one-way ANOVA followed by Tukey *post hoc* test. Data among groups at different concentrations were compared by two-way ANOVA, followed by Tukey *post hoc* test.

Based on the aforementioned evidence, to explore further the interconnections between IGF2BP2, DANCR, and PID1, we transduced U251 and U251-Eto cells with lentivirus-carried pHAGE-IGF2BP2, pHAGE-PID1, and pHAGE-DANCR alone or in combination. Western blot analysis and RT-qPCR (Figures 7A,B) confirmed the mimicking effect of pHAGE-IGF2BP2 and pHAGE-PID1, whereas overexpression of IGF2BP2 resulted in reduced PID1 expression. Meanwhile, cells treated with IGF2BP2 or IGF2BP2 + PID1 exhibited upregulated expression of DANCR and downregulated expression of FOXO1, confirming previous findings that IGF2BP2 stabilizes DANCR expression and inhibits FOXO1.

MTT assay data showed that overexpression of IGF2BP2 resulted in elevated viability and IC_50_ of GBM cells, whereas overexpression of PID1 reversed the effect caused by IGF2BP2 (Figures 7C,D). In addition, PID1 upregulation increased cell apoptosis and decreased cell survival, stimulating the cytotoxic effect of etoposide and inhibiting the effect induced by IGF2BP2 or DANCR upregulation (Figures 7E,F; Supplementary Figure S1C). These results indicated that IGF2BP2 diminished the sensitivity of GBM cells to etoposide by inhibiting the expression of PID1.

Finally, we verified the effect of IGF2BP2 and PID1 *in vivo* on the resistance of GBM cells. Injection of PID1-overexpressing cells decreased Ki67 protein level in the tumors and increased the survival rate of the nude mice. Combined treatment of IGF2BP2 + PID1 or DANCR + PID1 elevated Ki67 protein expression, but diminished the survival rate of nude mice (Supplementary Figures S4A–C; Supplementary Table S3). Taken together, these lines of evidence indicated that IGF2BP2 promoted etoposide resistance in GBM cells through the DANCR/PID1 axis.

## Discussion

Glioma remains a major challenge in clinical oncology where several contributing factors have hindered efforts to improve outcomes, notably the high rates of *de novo* and acquired chemoresistance (Reardon and Wen, 2015). Recent advances have improved our understanding of glioma pathogenesis, and several clinically significant genetic alterations have been described (Lapointe et al., 2018). In this study, we identified IGF2BP2 as a potential target to prevent the chemoresistance of GBM, as the evidence indicated that IGF2BP2 promoted etoposide resistance in GBM through the DANCR/PID1/FOXO1 axis.

IGF2BP2 (Imp2) preserves GBM stem cells, which tend to drive cancer progression by preventing the silencing of the let-7 target gene (Degrauwe et al., 2016). Besides, IGF2BP2 regulates oxidative phosphorylation in primary GBM, whereas depletion of Imp2 in gliomaspheres decreases their oxygen consumption rate, resulting in impaired clonogenicity *in vitro* and tumorigenicity *in vivo* (Janiszewska et al., 2012). In line with previous studies, we confirmed that overexpression of IGF2BP2 reduced etoposide-induced apoptosis and increased GBM cell survival. Furthermore, the evidence showed that IGF2BP2 induces etoposide resistance through DANCR regulation, whereas IGF2BP2 promoted DANCR stability and expression in GBM cells. Previously, an interaction between IGF2BP2 and DANCR has been implicated in pancreatic cancer, where IGF2BP2 promotes DANCR expression and DANCR contributes to tumorigenesis and cancer cell growth (Hu et al., 2020). DANCR, an important cancer-related lncRNA (Thin et al., 2018), is known to promote cell growth in glioma and induce drug resistance through certain signaling pathways or sponging microRNAs (Ma et al., 2018; Xu et al., 2018; Feng et al., 2020). However, the mechanism underlying its role of drug resistance of glioma cells remains elusive. Glioma cases associated with better survival is characterized by the glioma CpG island methylator phenotype and aberrant methylation of particular genes (LeBlanc and Marra, 2016). Results of the RIP assay in the present study revealed that DANCR is modified by methylation in GBM cells, but that IGF2BP2 can still recognize methylated DANCR.

Furthermore, we identified FOXO1 as a DANCR target gene in the present study. FOXO transcription factors are critical mediators of apoptosis in cytotoxic drugs, and FOXO1 silencing attenuates intracellular reactive oxygen species levels, thereby leading to drug resistance through a downstream target (Goto and Takano, 2009). Forced FOXO1 suppresses the cell growth through G2/M cell cycle arrest and increases apoptosis in glioma (Piao et al., 2019), whereas silencing of DANCR could repress the ubiquitination of FOXO1 in osteoblast differentiation and reduce the amount of DANCR bound to FOXO1 (Tang et al., 2018). In this study, overexpression of FOXO1 decreased the viability of U251 and U251-Eto cells, accompanied with decreased IC_50_ of the drug etoposide, whereas additional treatment with overexpression of DANCR restored the cytotoxic effect induced by FOXO1. Furthermore, DANCR interacted with FOXO1 to promote the ubiquitination of FOXO1, thereby inhibiting the stability of FOXO1 protein levels. FOXO1 promoted expression of PID1, and the binding of FOXO1 to the PID1 promoter could activate the transcription of PID1 in glioma stem cell (Zhao et al., 2017). Besides, higher PID1 mRNA levels also correlated with longer overall survival in patients with glioma, whereas in cell culture, overexpression of PID1 inhibited colony formation and induced mitochondrial depolarization, as sign of oxidative stress (Erdreich-Epstein et al., 2014). In the presence of FOXO1, glioma cell proliferation is inhibited, but addition of PID1 allows the proliferation ability to return to control levels (Zhao et al., 2017). The small number of articles reporting on PID1 to date hardly clarifies the role of PID1 in drug resistance in glioma and its association with FOXO1. In this regard, our work consistently confirmed the binding relationship between PID1 and FOXO1 and demonstrated that silencing of PID1 inhibited etoposide-induced apoptosis of GBM lines, whereas the addition of overexpressed FOXO1 abrogated that effect, thus restoring apoptosis. Moreover, overexpression of PID1 also reduced the effect of overexpressed IGF2BP2 as upregulation of IGF2BP2 resulted in an elevated viability and high etoposide IC_50_ of GBM cells.

**FIGURE 8:**
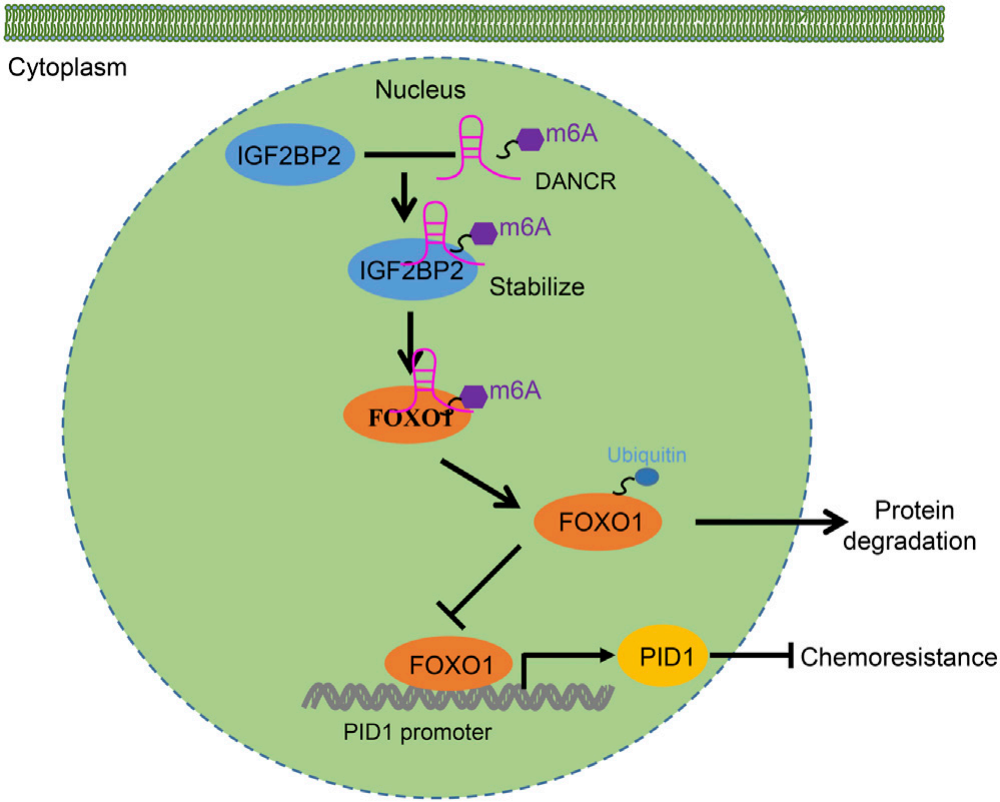
Schematic diagram of the mechanism by which IGF2BP2 affects the resistance of GBM cells to etoposide. IGF2BP2 promotes DANCR expression and stability through FOXO1 ubiquitination-mediated PID1 expression, thereby promoting cancer cell survival and tumor growth as well as engendering the etoposide resistance of GBM cells to etoposide.

In conclusion, this study elucidates a mechanism by which IGF2BP2 promotes DANCR expression and stability through FOXO1 ubiquitination-mediated PID1 expression, thereby promoting cancer cell survival and tumor growth as well as promoting the resistance of GBM cells to etoposide (Figure 8). These findings might contribute to the future development of novel target therapies for glioma. Further investigations are still required for the exploration of the specific molecular mechanism whereby DANCR affects the ubiquitination and degradation of FOXO1.

## Data Availability

The original contributions presented in the study are included in the article/Supplementary Material, further inquiries can be directed to the corresponding author.
