# Supplementary figures and images for: IGF2BP2 Induces U251 Glioblastoma Cell Chemoresistance by Inhibiting FOXO1-Mediated PID1 Expression Through Stabilizing lncRNA DANCR
Source: Front Cell Dev Biol. 2022 Jan 24;9:659228. doi: 10.3389/fcell.2021.659228 (PMC8819069; doi:10.3389/fcell.2021.659228)

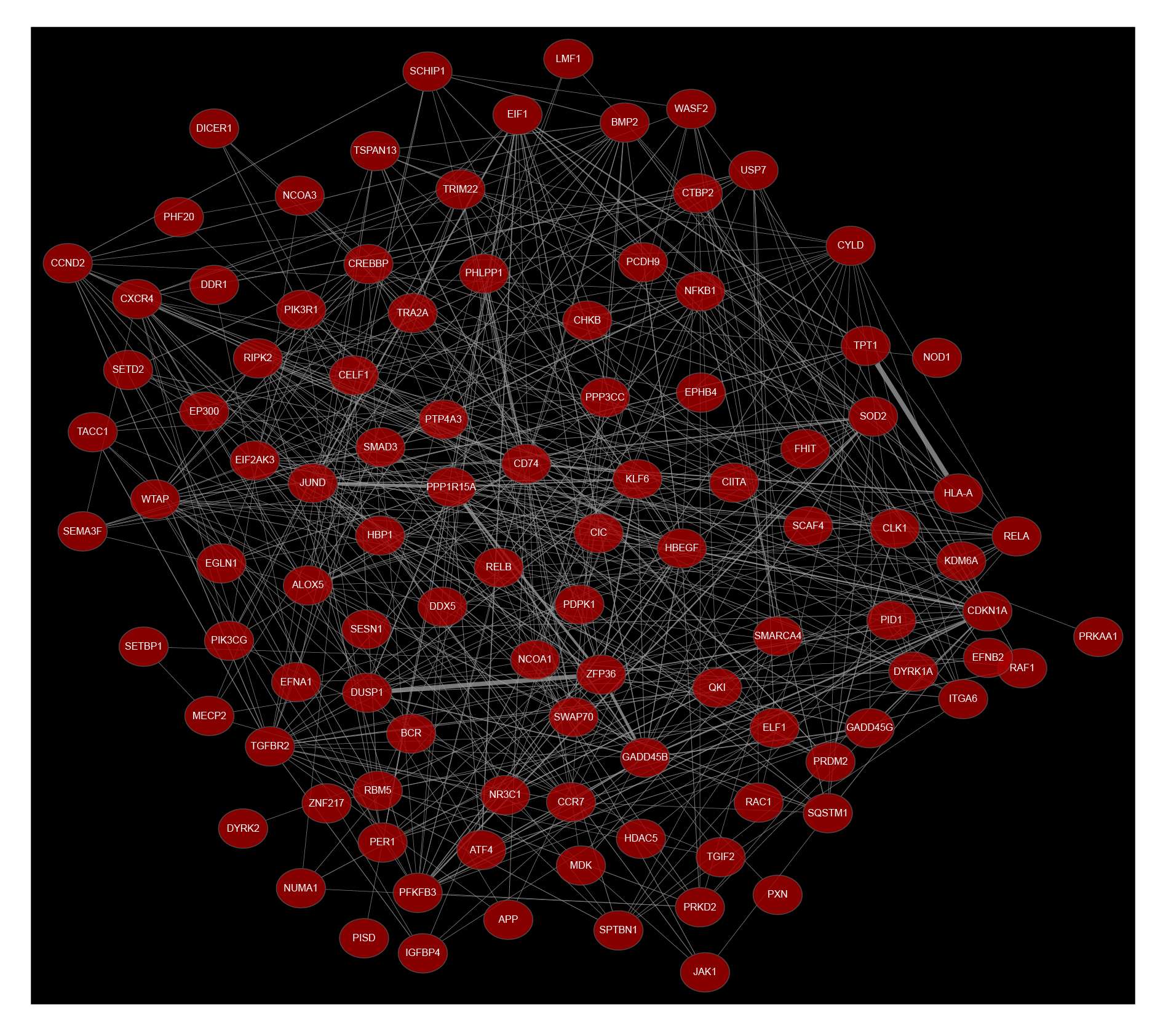

Supplement: Supplementary file 1 [file Image3.JPEG]

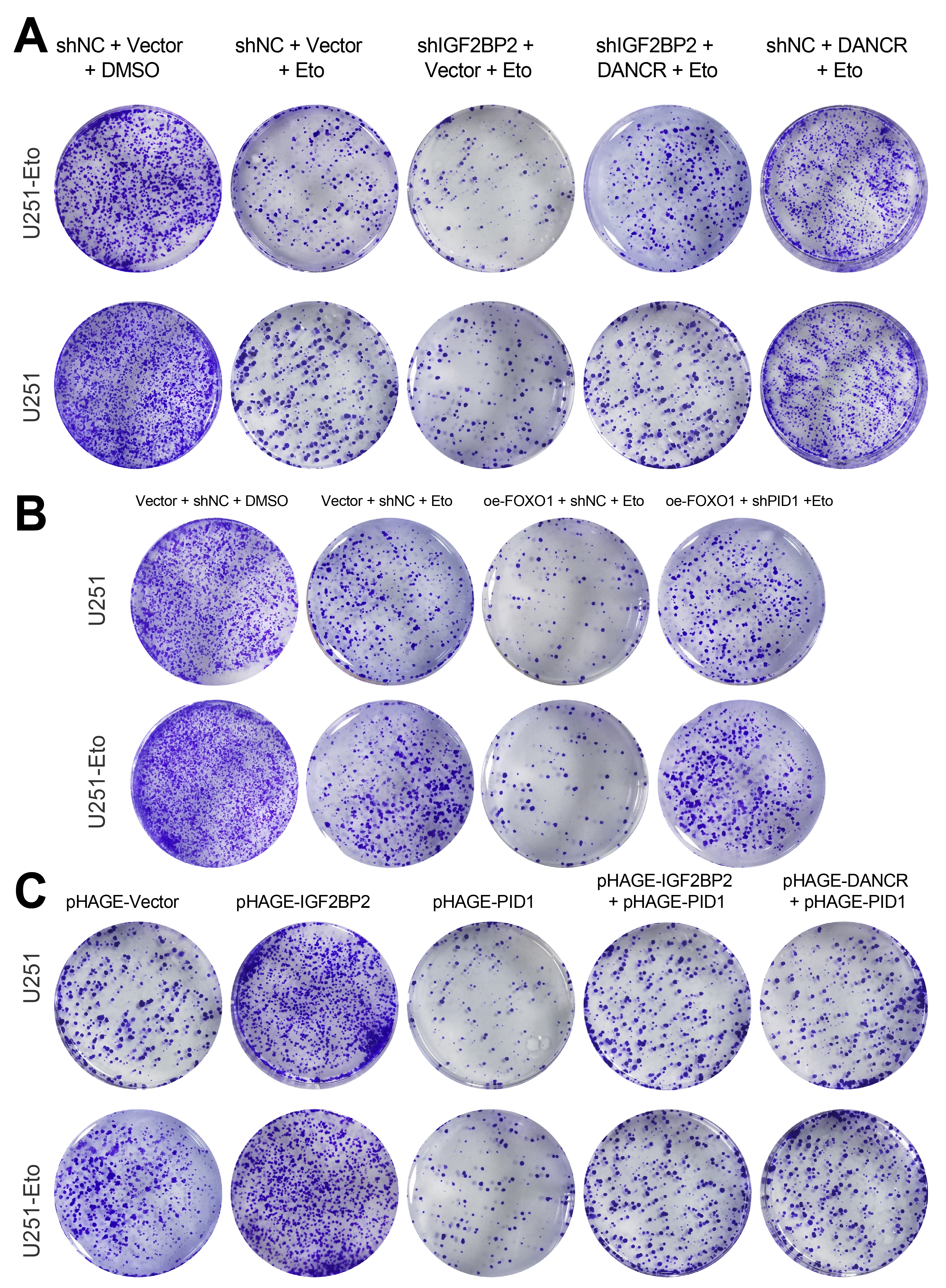

Supplement: Supplementary file 3 [file Image1.JPEG]

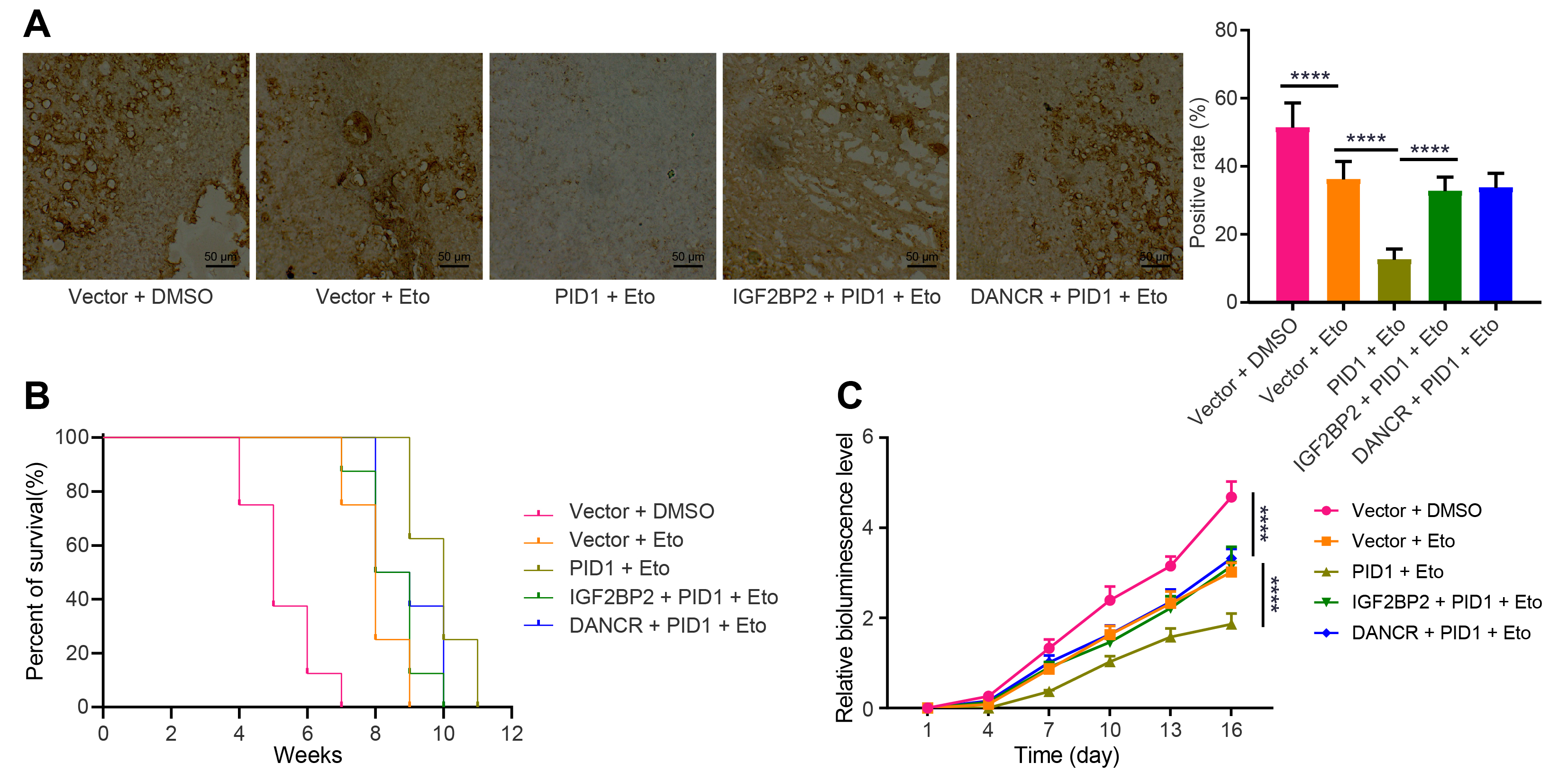

Supplement: Supplementary file 4 [file Image4.JPEG]

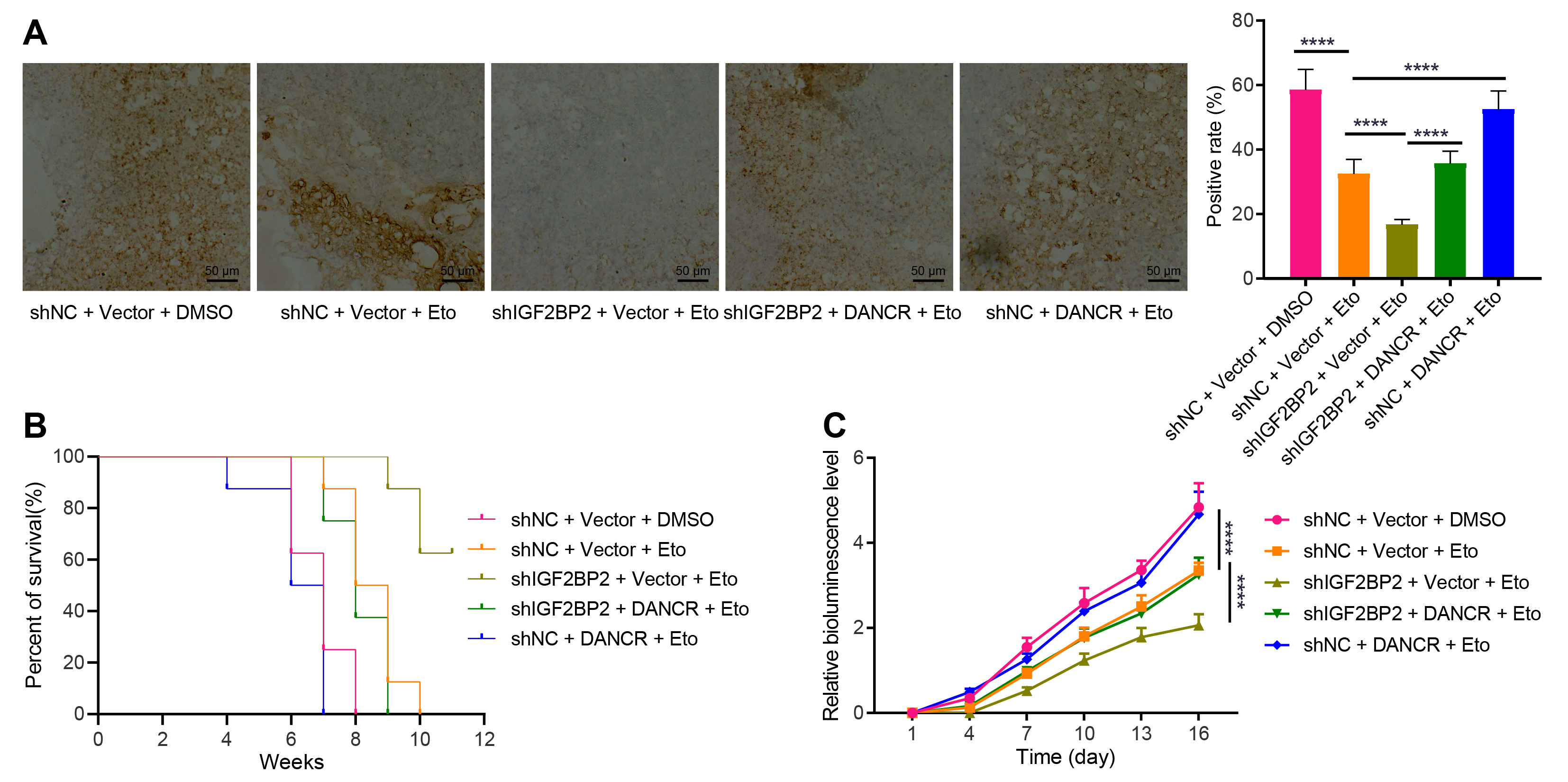

Supplement: Supplementary file 5 [file Image2.JPEG]
