# Supplementary material for: IGF2BP2 Induces U251 Glioblastoma Cell Chemoresistance by Inhibiting FOXO1-Mediated PID1 Expression Through Stabilizing lncRNA DANCR
Source: Front Cell Dev Biol. 2022 Jan 24;9:659228. doi: 10.3389/fcell.2021.659228 (PMC8819069; doi:10.3389/fcell.2021.659228)
Supplement: Supplementary file 2 [file Table1.DOCX]

**SUPPLEMENTARY TABLE 1 |** shRNA sequences

| shRNA | Sequence |
| --- | --- |
| sh-NC | 5’-TTCTCCGAACGTGTCACGT-3’ |
| Homo-sh-IGF2BP2-1 | 5’-GCTGGAAGCGCATATCAGA-3’ |
| Homo-sh-IGF2BP2-2 | 5’-ACCAAACTAGCCGAAGAGATT-3’ |
| Homo-sh-DANCR-1 | 5’-GGAGCTAGAGCAGTGACAATG-3’ |
| Homo-sh-DANCR-2 | 5’-GCGTACTAACTTGTAGCAA-3’ |
| Homo-sh-FOXO1-1 | 5’-GCTTAGACTGTGACATGGAAT-3’ |
| Homo-sh-FOXO1-2 | 5’-ATCTACGAGTGGATGGTCAA-3’ |
| Homo-sh-PID1-1 | 5’-GACCATGCTGAAGTCTAAATT-3’ |
| Homo-sh-PID1-2 | 5’-AGACTTTCCACAGTATGAAGA-3’ |

**SUPPLEMENTARY TABLE 2 |** Primer sequences for RT-qPCR

| Gene | Sequence |
| --- | --- |
| DANCR | F: 5’-GCCACTATGTAGCGGGTTTC-3’ |
|  | R: 5’-ACCTGCGCTAAGAACTGAGG-3’ |
| IGF2BP2 | F: 5’-ACGACCTCCGGCAGCTCTTTG-3’ |
|  | R: 5’-GGATGTTTCGAATCTGAAT-3’ |
| FOXO1 | F: 5’-GGCTACTCCTCCGTGAGCAG-3’ |
|  | R: 5’-CATGAGGTCATTCCGAATGATG-3’ |
| PID1 | F: 5’-GATTGCTGGCAACCACCTGATGT-3’ |
|  | R: 5’-AAATGTAAAGGTTGGCAGGGCCAC-3’ |
| GAPDH | F: 5’-AGAAGGCTGGGGCTCATTTG-3’ |
|  | R: 5’-AGGGGCCATCCACAGTCTTC-3’ |

**SUPPLEMENTARY TABLE 3 |** Median survival & standard deviation of panel B in Supplementary Figure 1 and Supplementary Figure 3

| Treatment | Median survival | Standard deviation |
| --- | --- | --- |
| shNC + Vector + DMSO | 7 | 0.83 |
| shNC + Vector + Eto | 8.5 | 0.93 |
| shIGF2BP2 + Vector + Eto | 11 | 0.76 |
| shIGF2BP2 + DANCR + Eto | 8 | 0.83 |
| shNC + DANCR + Eto | 6.5 | 1.04 |
| Vector + DMSO | 5 | 1.04 |
| Vector + Eto | 8 | 0.83 |
| PID1 + Eto | 10 | 0.83 |
| IGF2BP2 + PID1 + Eto | 8.5 | 0.93 |
| DANCR + PID1 + Eto | 8.5 | 0.99 |

**Note**: NC, negative control; sh-, short hairpin RNA; PID1, phosphotyrosine interaction domain containing 1
